# Placental Angiogenic Imbalance and Its Association With Adverse Outcomes in Congenital Heart Disease Pregnancies

**DOI:** 10.1016/j.jacadv.2025.102301

**Published:** 2025-11-04

**Authors:** Nour Rahnama, Arthur Colson, Agnès Pasquet, Damien Gruson, Julie Hotton, Nassiba Menghoum, David Vancraeynest, Christophe Beauloye, Frédéric Debiève, Sophie Pierard

**Affiliations:** a Cardiovascular Department, Cliniques Universitaires Saint-Luc, Brussels, Belgium; b Pôle de Recherche Cardiovasculaire (CARD), Institut de Recherche Expérimentale et Clinique (IREC), Université Catholique de Louvain (UCLouvain), Brussels, Belgium; c Obstetrics Department, Cliniques Universitaires Saint-Luc, Brussels, Belgium; d Physiopathologie de la Reproduction (REPR), Institut de Recherche Expérimentale et Clinique (IREC), Université Catholique de Louvain (UCLouvain), Brussels, Belgium; e Department of Laboratory Medicine, Cliniques Universitaires Saint-Luc, Brussels, Belgium; f Department of Laboratory Medicine, Cliniques de l’Europe, Brussels, Belgium

**Keywords:** congenital heart disease, placenta, PlGF, pregnancy, sFlt-1

## Abstract

**Background:**

Pregnancy in women with congenital heart disease (CHD) carries an increased risk of obstetric and neonatal complications. While the underlying mechanisms remain unclear, placental dysfunction has been hypothesized to contribute to these outcomes.

**Objectives:**

This study aimed to evaluate biomarkers of placental angiogenesis, soluble fms-like tyrosine kinase-1 (sFlt-1) and placental growth factor (PlGF), in pregnant women with CHD and assess their association with adverse pregnancy outcomes.

**Methods:**

This prospective study included 95 pregnant women, 32 with CHD and 63 healthy controls. Serum sFlt-1 and PlGF were measured during the third trimester, and the sFlt-1/PlGF ratio was calculated. In a subset of 66 patients with available first-trimester serum, we assessed longitudinal changes in biomarker levels between the first and third trimesters. Associations with obstetric and neonatal complications were evaluated.

**Results:**

Women with CHD had higher third-trimester sFlt-1 (2,578 pg/mL [25th-75th percentile: 2,095-4,493 pg/mL] vs 1,983 pg/mL [25th-75th percentile: 1,414-2.788 pg/mL], *P* = 0.002) and lower PlGF (276 pg/mL [25th-75th percentile: 164-510 pg/mL] vs 515 pg/mL [25th-75th percentile: 289-752 pg/mL], *P* = 0.005) than controls, resulting in an elevated sFlt-1/PlGF ratio (9.55 [25th-75th percentile: 3.88-26.70]) vs 3.83 [25th-75th percentile: 1.91-7.32], *P* = 0.001). CHD patients with high sFlt-1 had a 4-fold higher risk of adverse outcomes than those with normal levels (80% vs 22.2%; *P* = 0.024). The rise in sFlt-1 between the first and third trimesters was greater in CHD patients than in controls (*P* = 0.008).

**Conclusions:**

Pregnant women with CHD exhibited a placental angiogenic imbalance, with higher sFlt-1 and lower PlGF in late pregnancy than controls. This imbalance was associated with a higher risk of adverse pregnancy outcomes, particularly in women with high sFlt-1.

Pregnancy in women with congenital heart disease (CHD) is associated with an increased risk of adverse obstetric and neonatal outcomes, including preterm birth, preeclampsia, and fetal growth restriction (FGR), compared to the general population.^1^, ^2^, ^3^, ^4^ While the exact mechanisms underlying these complications are not yet fully understood, growing evidence suggests that placental dysfunction may be the primary contributing factor.^5^, ^6^, ^7^, ^8^, ^9^ In our previous study, we prospectively demonstrated a higher prevalence of maternal vascular malperfusion in pregnant women with CHD compared to a control group, which was associated with adverse pregnancy outcomes.^10^

Research on preeclampsia has shown that placental dysfunction leads to an alteration of the placental angiogenic balance, characterized by elevated levels of soluble fms-like tyrosine kinase-1 (sFlt-1) and reduced levels of placental growth factor (PlGF).^11^, ^12^, ^13^ sFlt-1 is an antiangiogenic protein that acts by binding and sequestering vascular endothelial growth factor (VEGF) and PlGF, preventing them from interacting with their receptors on the endothelium.^14^, ^15^, ^16^ This results in reduced angiogenesis and impaired placental vascular development.^17^ PlGF, on the other hand, is a pro-angiogenic factor that promotes blood vessel formation, which is crucial for adequate placental function and fetal development.^18^ An increased sFlt-1/PlGF ratio has been linked to complications such as preeclampsia, FGR, and other placental dysfunction-related disorders.^19^

This study aimed 1) to determine whether women with CHD exhibit biological markers of placental dysfunction by comparing their serum levels of sFlt-1 and PlGF to those of a control group, and 2) to assess the association between these biomarkers and adverse obstetric and neonatal outcomes in this population.

## Methods

### Study population

This study included a subset of women who participated in our previous investigation on placental abnormalities in pregnancies affected by CHD, compared with a control group of pregnancies without CHD.^10^ From the original cohort, women for whom serum samples were collected during the third trimester were included in the primary analysis of this study.

A secondary analysis was conducted on a subset of these patients for whom first-trimester serum samples were also available, in order to assess changes in biomarker levels over time. The availability of first-trimester serum samples was affected by several factors. Fifteen patients were screened during the first trimester but were officially recruited only in the second trimester due to delays in obtaining ethics committee approval, preventing blood draws before consent was signed. Nine patients declined first-trimester blood draws due to the already high number of pregnancy-related tests, agreeing to participate only if a single blood draw was conducted. Additionally, organizational challenges early in the study (n = 5), such as missed appointments, also contributed to missing samples.

The inclusion and exclusion criteria were consistent with those outlined in our previous study.^10^ All participants were pregnant women ≥18 years of age, recruited during the first trimester (6 + 0 to 13 + 6 weeks of gestation) from the adult congenital heart disease consultation and the obstetrics consultation at Cliniques Universitaires Saint-Luc. High-risk pregnancies were excluded from both groups. This included women with chronic inflammatory or infectious disease, immunosuppression, multiple gestation, or a history of preeclampsia, FGR, or preterm birth in previous pregnancies. Additionally, women who smoked or consumed alcohol during pregnancy were excluded. To ensure comparability between groups, participants with and without CHD were matched for maternal age, body mass index, and parity.

The study protocol was approved by our Institutional Review Board (Comité d'éthique hospitalo-facultaire Saint-Luc—UCL, Reference: 2020/09DEC/612, Belgian Registration Number: BE4032020000120), and written informed consent was obtained from all participants.

### Pregnancy outcomes

Adverse pregnancy outcomes were defined as the occurrence of at least one of the following obstetric or neonatal complications during pregnancy: hypertensive disorders of pregnancy (HDPs), including pregnancy-induced hypertension (PIH) (new onset hypertension with blood pressure ≥140 mm Hg systolic or ≥90 mm Hg diastolic without proteinuria after 20 weeks of gestation) and preeclampsia (PIH with one of the following: ≥0.3 g protein in a 24-hour urine sample, maternal organ dysfunction, or FGR), placental abruption, threatened preterm labor (uterine contractions before 37 weeks of gestation requiring tocolysis), preterm birth (birth before 37 weeks’ gestation), and small-for-gestational-age (newborn’s weight below the 10th percentile).

### sFlt-1 and PlGF assay

Blood samples were collected during the first and third trimester of pregnancy (between 27 + 0 and 37 + 6 weeks of gestation) for the measurement of sFlt-1 and PlGF. Following collection, the blood samples were centrifuged at 1.64 g for 10 minutes to separate the serum, which was then aliquoted and stored at −80 °C until analysis. We measured sFlt-1 and PlGF levels using a two-site automated electrochemiluminescence assay on the Cobas 8000 platform (Roche Diagnostics). For each patient, the sFlt-1/PlGF ratio was calculated to assess the angiogenic balance.

### Statistical analysis

Continuous variables are presented as median (25th–75th percentile) and categorical variables as counts and percentages. Group comparisons were performed using the Mann-Whitney *U* test for continuous variables and the chi-square or Fisher exact test for categorical variables. Correlations between continuous variables were assessed using Pearson’s or Spearman’s correlation coefficients, as appropriate. A 2-tailed value of *P* < 0.05 was considered statistically significant.

Due to the non-normal distribution of the biomarker data, sFlt-1, PlGF, and the sFlt-1/PlGF ratio were log-10 transformed to normalize their distributions and stabilize variance. sFlt-1 levels were considered high if they exceeded the control group mean + 2 SD of the log-transformed values (>5,400.3 pg/mL in the original scale). The same method was applied to identify the threshold for a high sFlt-1/PlGF ratio (>30.5). PlGF levels were classified as low if they were below the control group mean − 2 SD of the log-transformed values (<104.4 pg/mL in the original scale).

Longitudinal changes in biomarker levels between the first and third trimesters were assessed by calculating both the absolute change (Δ, third trimester levels-first trimester levels) and the relative change (ratio, third trimester levels/first trimester levels) for each patient. In addition, to formally test whether biomarker trajectories differed between groups, we fitted linear mixed-effects models with fixed effects for group (CHD vs control), time (first vs third trimester), and their interaction, and a random intercept for each subject.

Because biomarker levels show the greatest gestational variation in the third trimester, additional analyses were performed to account for the influence of gestational age at the time of sampling. Linear regression models were fitted with log-transformed biomarker values as the dependent variable, group (CHD vs control) as the main predictor, and gestational age at blood draw as a covariate. In addition, sensitivity analyses were performed using 1:1 nearest-neighbor matching of CHD patients and controls based on gestational age at third-trimester sampling.

All analyses were conducted using R (R Core Team, 2020) and RStudio (Rstudio Team, 2020).

## Results

### Baseline characteristics and pregnancy outcomes

**Figure 1:**
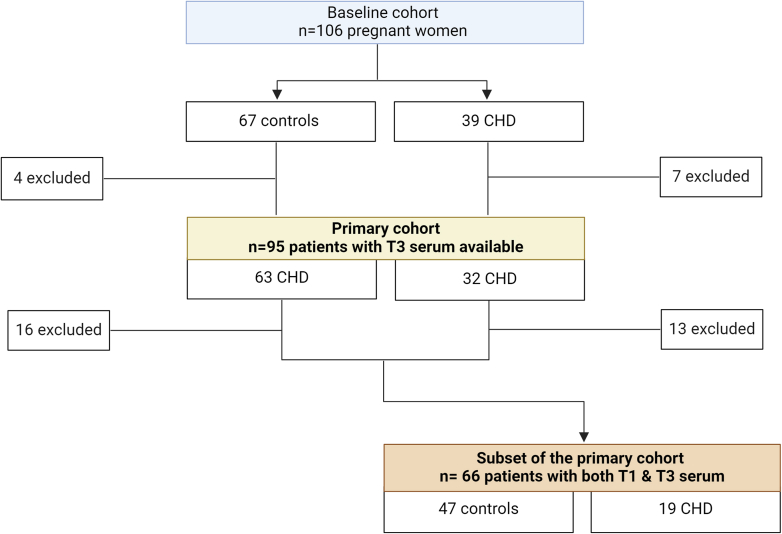
**Flowchart of the Study Population** The initial cohort consisted of 106 pregnant women, including 67 controls and 39 women with CHD. After excluding women with missing third-trimester serum samples (n = 11), 95 women (63 controls and 32 CHD patients) were included in the primary analysis, which focused on third-trimester biomarker levels of sFlt-1 and PlGF. A subanalysis was conducted in a subset of 66 women (47 controls and 19 CHD patients) for whom both first and third trimester serum samples were available. A total of 29 women (16 controls and 13 CHD patients) were excluded from this subanalysis due to missing first-trimester serum samples. T1 = first trimester; T3 = third trimester; CHD = congenital heart disease; PlGF = placental growth factor; sFlt-1 =soluble fms-like tyrosine kinase-1.

Among the 106 women included in the study, 95 (63 controls and 32 CHD patients) had serum samples collected during the third trimester and were included in this analysis. First-trimester serum samples were further analyzed in a subset of 66 women (47 controls and 19 CHD patients) to assess biomarker changes over time (Figure 1).

In the 32 CHD patients, atrial septal defects, ventricular septal defects, and tetralogy of Fallot were the most common lesions, together accounting for 62.5% of the CHD cases in the cohort. The complexity of the lesions was classified according to the European Society of Cardiology guidelines, with all cases being of mild or moderate complexity, except for one case of complex CHD. The distribution of the different types of CHD lesions and their complexity is provided in Table 1.

**Table 1:** Distribution of CHD Lesion Types and Complexity in the Study Cohort (N = 32)

CHD lesion type	
ASD	9 (28.1)
VSD	5 (15.6)
ToF	4 (12.5)
PS	4 (12.5)
TGA	3 (9.4)
Ebstein	2 (6.2)
AVD	2 (6.2)
CoA	2 (6.2)
PAVSD	1 (3.1)
CHD complexity^a^	
Mild	15 (46.9)
Moderate	16 (50.0)
Severe	1 (3.1)

Values are n (%).

ASD = atrial septal defect; AVD = aortic valve dysplasia; CHD = congenital heart disease; CoA = coarctation of the aorta; PAVSD = pulmonary atresia with ventricular septal defect; PS = pulmonary stenosis; TGA = transposition of the great arteries; ToF = tetralogy of Fallot; VSD = ventricular septal defect.

a CHD complexity was classified according to the European Society of Cardiology (ESC) guidelines.

Baseline characteristics of the 95 patients were similar between the CHD and control groups, with the exception of a higher rate of in vitro fertilization in the CHD group (*P* = 0.016) and a higher prepregnancy smoking rate among CHD patients (*P* = 0.005) (Table 2), but without active smoking during pregnancy. Additionally, the pregnancy risk profile was similar between the 2 groups, with no significant differences observed in factors such as maternal age, gestational diabetes, or weight gain during pregnancy (Table 2).

**Table 2:** Baseline Characteristics of Patients

	All (N = 95)	Controls (n = 63)	CHD (n = 32)	*P* Value
Maternal age at pregnancy, y	30 (27–32)	30 (27–32)	30 (27–33)	0.740
Primiparous	54 (56.8)	37 (58.7)	17 (53.1)	0.763
In vitro fertilization	6 (6.3)	1 (1.6)	5 (15.6)	**0.016**
Prepregnancy CVRF				
Hypertension	2 (2.1)	1 (1.6)	1 (3.1)	1.000
Diabetes	0 (0.0)	0 (0.0)	0 (0.0)	
History of smoking	7 (7.4)	1 (1.6)	6 (18.8)	**0.005**
Obesity (BMI ≥30 kg/m^2^)	14 (14.7)	8 (12.7)	6 (18.8)	0.542
Baseline clinical parameters^a^				
Systolic blood pressure, mm Hg	120 (111–126)	120 (112–125)	121 (110–126)	0.812
Diastolic blood pressure, mm Hg	71 (65–79)	71 (64–77)	76 (67–80)	0.243
SpO_2_, %	98 (97–98)	98 (97–99)	98 (97–98)	0.205
BMI, kg/m^2^	24 (22–27)	23 (21–27)	2.5 (22.5–27)	0.206
Pregnancy risk factors				
Age >35 y	9 (9.5)	6 (9.5)	3 (9.4)	1.000
Active smoking during pregnancy	0 (0.0)	0 (0.0)	0 (0.0)	
Gestational diabetes	11 (11.6)	6 (9.5)	5 (15.6)	0.499
Total weight gain, kg	9 (6–11.5)	9.5 (6.4–11.4)	8.7 (6–11.8)	0.592

Values are median (25th-75th percentile) or n (%), unless otherwise indicated. **Bold** indicates statistically significant differences (*P* < 0.05).

BMI = body mass index; CVRF = cardiovascular risk factor; other abbreviation as in Table 1.

a Parameters at first trimester of pregnancy (inclusion).

Pregnancy outcomes are summarized in Table 3. The CHD group had a significantly lower median gestational age at term compared to the control group (39.0 weeks [25th-75th percentile: 38.2-39.7] vs 39.4 weeks [25th-75th percentile: 38.8-40.2], *P* = 0.009). Overall, women with CHD exhibited a higher prevalence of complications compared to controls (31.2% vs 7.9%; *P* = 0.008). The most common complications in the CHD group were HDPs (n = 6) (including 4 PIH and 2 preeclampsia) and threatened preterm labor (n = 4).

**Table 3:** Pregnancy Outcomes

	All (N = 95)	Controls (n = 63)	CHD (n = 32)	*P* Value
Gestational age at term, wk	39.3 (38.5–40.2)	39.4 (38.8–40.2)	39.0 (38.2–39.7)	**0.009**
Newborn’s weight, g	3,305 (3,058–3,602)	3,315 (3,080–3,595)	3,300 (3,041–3,598)	0.674
Pregnancy outcomes	15 (15.8)	5 (7.9)	10 (31.2)	**0.008**
HDPs	7 (7.4)	1 (1.6)	6 (18.8)	
Placental abruption	2 (2.1)	1 (1.6)	1 (3.1)	
Threatened preterm labor	6 (6.3)	2 (3.1)	4 (12.5)	
Preterm birth	2 (2.1)	0 (0.0)	2 (6.25)	
SGA	3 (2.8)	3 (4.5)	0 (0.0)	

Values are median (25th-75th percentile) or n (%), unless otherwise indicated. **Bold** indicates statistically significant differences (*P* < 0.05).

HDPs = hypertensive disorders of pregnancy; SGA = small-for-gestational age; other abbreviation as in Table 1.

### Third trimester biomarker levels of sFlt-1 and PlGF in CHD and control patients

**Figure 2:**
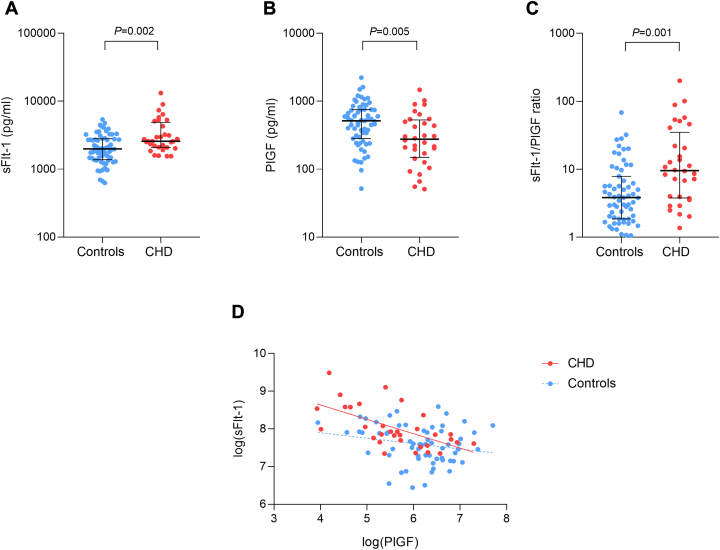
**Placental Angiogenic Biomarkers in CHD vs Non-CHD Pregnant Women** (A) Serum sFlt-1 levels in CHD patients vs controls. (B) Serum PlGF levels in CHD patients vs controls. (C) sFlt-1/PlGF ratio in CHD patients vs controls. Error bars represent median and IQR. (D) Scatter plot showing the inverse correlation between log-transformed sFlt-1 and PlGF levels in both groups. The correlation was stronger in the CHD group (red) than in controls (blue) Abbreviations as in Figure 1.

Blood sampling in the third trimester was performed at a median gestational age of 32.5 weeks (25th-75th percentile: 31.2–33.8 weeks) in controls and 34.7 weeks (25th-75th percentile: 33.2–36.0 weeks) in CHD patients. sFlt-1 levels were significantly higher in the serum of CHD patients compared to controls (2,578 pg/mL [25th-75th percentile: 2,095-4,493 pg/mL] vs 1,983 pg/mL [25th-75th percentile: 1,414-2,788 pg/mL], *P* = 0.002) (Figure 2A). All patients with high sFlt-1 levels (n = 5) belonged to the CHD group. In contrast, PlGF levels were significantly lower in the CHD group (276 pg/mL [25th-75th percentile: 164-510 pg/mL] vs 515 pg/mL [25th-75th percentile: 289-752 pg/mL], *P* = 0.005) (Figure 2B). Six of the 8 patients with low PlGF levels belonged to the CHD group. We observed an increased sFlt-1/PlGF ratio among CHD patients (9.55 [25th-75th percentile: 3.88-26.7]) compared to controls (3.83 [25th-75th percentile: 1.91-7.32], *P* = 0.001) (Figure 2C). The majority of patients with a high sFlt-1/PlGF ratio (8 out of 10) belonged to the CHD group.

After adjustment for gestational age at third-trimester sampling, the differences in sFlt-1, PlGF, and the sFlt-1/PlGF ratio remained significant (Supplemental Tables 1 and 2). Sensitivity analyses excluding patients with a history of prepregnancy smoking or in vitro fertilization yielded consistent results, with all comparisons remaining statistically significant (all *P* < 0.05, Supplemental Table 3).

A negative correlation between PlGF and sFlt-1 was observed in both groups (Figure 2D). In the control group, this correlation was weak (*r* = −0.21, *P* = 0.097), whereas a stronger and statistically significant inverse correlation was identified in the CHD group (*r* = −0.61, *P* < 0.001).

### Association between third trimester sFlt-1 and PlGF and pregnancy outcomes in CHD patients

**Figure 3:**
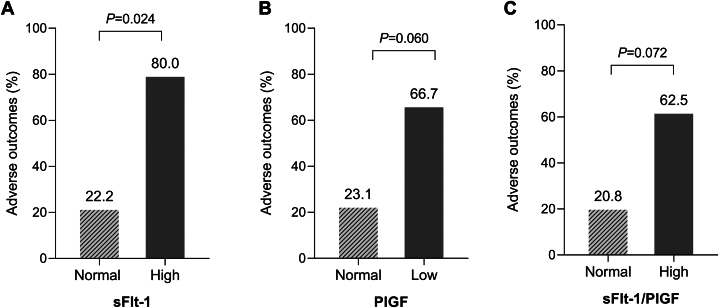
**Association Between Third-Trimester Biomarkers and Adverse Outcomes in CHD Pregnancies** (A) CHD women with high sFlt-1 levels (n = 5) experienced a significantly higher percentage of adverse pregnancy outcomes compared to those with normal sFlt-1 levels (n = 27). (B) CHD women with low PlGF levels (n = 6) showed a trend toward more adverse outcomes compared to those with normal PlGF levels (n = 26). (C) CHD women with a high sFlt-1/PlGF ratio (n = 8) also showed a trend toward more adverse outcomes compared to those with a normal ratio (n = 24) Abbreviations as in Figure 1.

Thirty-one percent of women with CHD experienced obstetric or neonatal complications during pregnancy. CHD women with high sFlt-1 levels had higher risk of adverse pregnancy outcomes compared to those with non-elevated sFlt-1 levels (80.0% vs 22.2%; *P* = 0.024; Figure 3A). Additionally, patients with low PlGF levels showed a trend toward increased adverse events compared to CHD women with non-reduced PlGF (66.7% vs 23.1%; *P* = 0.060; Figure 3B), and those with a high sFlt-1/PlGF ratio also demonstrated a similar trend (62.5% vs 20.8%; *P* = 0.072; Figure 3C).

The sFlt-1/PlGF ratio was also inversely correlated with newborn weight in the CHD group (*r* = −0.44, *P* = 0.011) (Supplemental Figure 1).

### Evolution of sFlt-1 and PlGF levels during pregnancy in women with CHD vs controls

**Figure 4:**
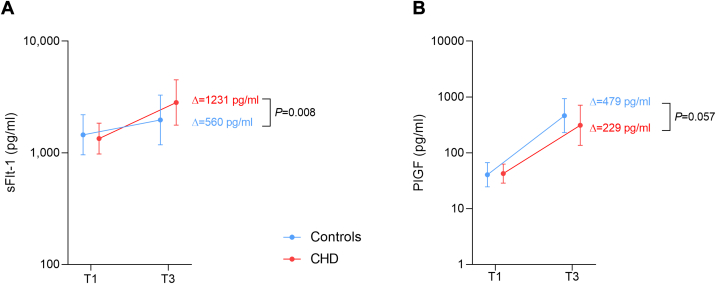
**sFlt-1 and PlGF Changes During Pregnancy in CHD Vs Controls** (A) sFlt-1 levels increased significantly more in women with CHD (n = 19) compared to controls (n = 47) from the first trimester (T1) to the third trimester (T3), with a steeper rise observed in the CHD group. (B) PlGF levels tended to increase less in CHD patients compared to controls between T1 and T3, although this difference did not reach statistical significance. Data are presented as mean values (points), with error bars representing SDs. Abbreviations as in Figure 1.

First-trimester serum was available in a subgroup of 66 patients, with blood sampling performed at a median gestational age of 12.3 weeks (25th-75th percentile: 11.4–12.8 weeks) in controls and 11.6 weeks (25th-75th percentile: 11.2–12.1) weeks in CHD patients. Biomarker levels did not differ between groups early in pregnancy. However, the absolute changes in biomarker levels between the first and third trimesters differed significantly between groups (Table 4). sFlt-1 increased significantly more during pregnancy in women with CHD compared to controls (*P* = 0.008) (Figure 4A). Conversely, PlGF tended to increase less in women with CHD than in controls (*P* = 0.057) (Figure 4B). Significant group × time interactions were observed for sFlt-1 (β = 0.19, *P* = 0.002) and PlGF (β = −0.19, *P* = 0.038), indicating that biomarker trajectories differed between CHD and controls (Supplemental Table 4).

**Table 4:** Comparison of First-Trimester Biomarker Levels and Longitudinal Changes Between the First and Third Trimester in CHD and Control Women

	All (N = 66)	Controls (n = 47)	CHD (n = 19)	*P* Value
sFlt-1, pg/mL	1,432 (1,096–1833)	1,475 (114–1876)	1,293 (1,094–1,063)	0.336^a^
PlGF, pg/mL	44.2 (31.1–55.5)	44.7 (31.1–54.5)	42.3 (34.0–56.0)	0.944^a^
sFlt-1/PlGF ratio	35.6 (25.1–42.7)	38.2 (27.7 –42.5)	29.8 (21.1–45.9)	0.424^a^
ΔsFlt-1, pg/mL	736 (130–1,478)	560 (−76.50 to 1,362)	1,231 (672–2,001)	**0.008**
ΔPlGF, pg/mL	412 (195–728)	479 (218–742)	229 (167–474)	0.057

PlGF = placental growth factor; sFlt-1 = soluble fms-like tyrosine kinase-1; other abbreviation as in Table 1.

Values are median (25th-75th percentile), unless otherwise indicated. **Bold** indicates statistical significance. **Δ**: absolute change in biomarker levels between the first and the third trimester.

a *P* =values are from Mann-Whitney tests performed on log-transformed values.

**Figure 5:**
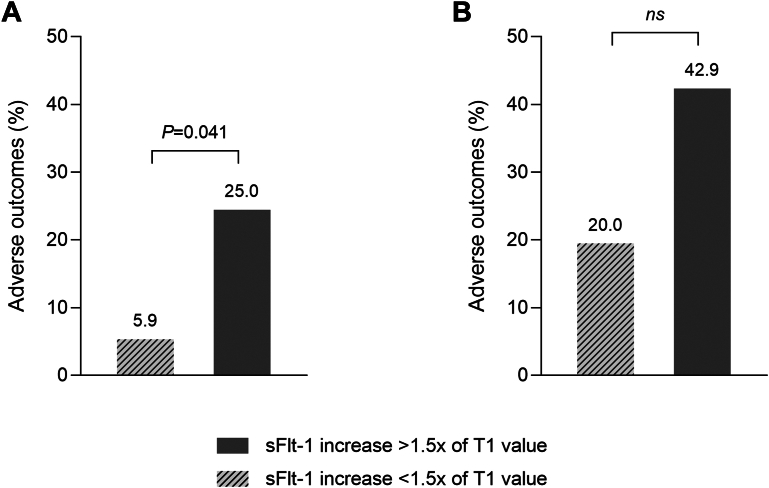
**Association Between sFlt-1 Increase During Pregnancy and Adverse Pregnancy Outcomes** (A) In the entire cohort (n = 66), patients whose sFlt-1 levels increased by more than 1.5 times their first-trimester value experienced a significantly higher rate of adverse outcomes compared to those with smaller increases. (B) In the CHD group (n = 19), although not statistically significant, patients with a more than 1.5-fold increase in sFlt-1 levels showed a higher rate of adverse outcomes compared to those with smaller increases. Ns = not significant; other abbreviations as in Figure 1.

Patients with a more than 1.5-fold increase in sFlt-1 levels (n = 32) experienced more complications compared to those with smaller increases (25% vs 5.9%; *P* = 0.041) (Figure 5A). However, this association was no longer significant when the analysis was restricted to the CHD group (n = 19) despite a doubling in the number of complications when sFlt-1 levels increased by more than 1.5-fold (Figure 5B). No significant association was found between relative change in PlGF levels and adverse outcomes.

## Discussion

To our knowledge, this study provides the first investigation into placental angiogenic biomarkers, specifically sFlt-1 and PlGF, in pregnant women with CHD compared to a control group. Our results can be summarized as follows:

1. Women with CHD exhibited angiogenic imbalance during the third trimester of pregnancy compared to controls, with higher sFlt-1 and lower PlGF levels in maternal serum.
2. High third-trimester sFlt-1 levels were associated with a four-fold increased risk of adverse obstetric and neonatal outcomes within the CHD group.
3. There was no evidence of angiogenic imbalance in women with CHD compared to controls during the first trimester of pregnancy.
4. The relative increase in sFlt-1 between the first and the third trimester was greater in the CHD group compared to the controls and an increase of more than one and a half times the baseline value was associated with a four-fold increase in obstetric and neonatal complications across the cohort.

### Placental angiogenic imbalance during the third trimester of pregnancy in women with CHD

We analyzed third-trimester serum samples from 32 pregnant women with predominantly mild or moderate CHD and 63 women without CHD. We found that CHD patients had significantly higher sFlt-1 and lower PlGF levels compared to controls, resulting in an elevated sFlt-1/PlGF ratio (Central Illustration). This angiogenic imbalance has been demonstrated by several authors in patients with placental dysfunction-related disorders, including preeclampsia, FGR, preterm birth, and placental abruption.^19^, ^20^, ^21^, ^22^, ^23^ During pregnancy, the placenta secretes VEGF-A and PlGF, glycoproteins promoting the proliferation, migration, and activation of endothelial cells.^24^ Both bind to the VEGF receptor-1, also called fms-like tyrosine kinase 1 (Flt-1), which activates pro-angiogenic signaling pathways.^24^ sFlt-1 is a variant of Flt-1 generated by an alternative splicing of the *FLT1* gene, lacking the transmembrane and intracellular tyrosine kinase domain. The latter acts as a decoy receptor by binding to free VEGF-A and PlGF, thereby reducing their availability for normal placental angiogenesis.^14^^,^^16^^,^^25^ In preeclampsia, the stressed syncytiotrophoblast is responsible for an overproduction of sFlt-1, leading to an excessive rise in maternal serum sFlt-1 levels starting from the 21st to 24th week of gestation, compared to women without preeclampsia.^25^, ^26^, ^27^ A corresponding decrease in PlGF serum levels was observed simultaneously.^26^ In our study, we found a stronger negative correlation between sFlt-1 and PlGF levels in the CHD group than in the control group, suggesting that the observed decrease in PlGF is linked to dysregulated placental production of sFlt-1 in CHD patients, similar to what occurs in preeclampsia. This is further supported by our subanalysis, which showed that sFlt-1 levels were similar between groups early in pregnancy but increased significantly more in the CHD than in the control group as pregnancy progressed. Combined with our previous results showing increased placental maternal vascular malperfusion in CHD pregnancies, these results provide further support for the hypothesis of placental dysfunction in this population.

**Central Illustration:**
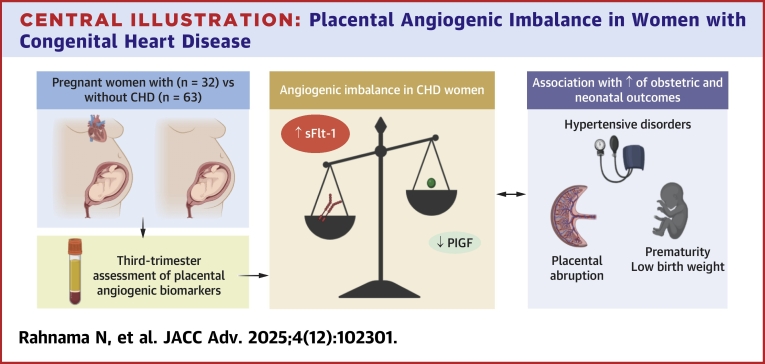
**Placental Angiogenic Imbalance in Women with Congenital Heart Disease** Pregnant women with CHD showed higher third-trimester sFlt-1 and lower PlGF levels compared to controls, resulting in an angiogenic imbalance. This imbalance was associated with an increased risk of obstetric and neonatal complications, supporting placental dysfunction as a key mechanism underlying adverse outcomes in CHD pregnancies. Abbreviations as in Figure 1. Figure created with BioRender.com.

However, the initial trigger of this altered placental angiogenesis remains uncovered. The relationship between cardiovascular health and placental function is increasingly recognized as both bidirectional and complex. On the one hand, maternal cardiac dysfunction can compromise placental development. In women with CHD, impaired uteroplacental Dopplers have been linked to reduced left ventricular function, lower cardiac output, and right ventricular dysfunction with venous congestion.^5^^,^^8^^,^^28^^,^^29^ Decline in maternal cardiac output has been associated with abnormal umbilical artery Doppler indices and adverse neonatal outcomes.^28^ Right ventricular dysfunction, reflected by reduced tricuspid annular plane systolic excursion, has similarly been related to increased uterine artery resistance as early as the first trimester,^29^ and abnormal uteroplacental Dopplers have been reported in women with repaired tetralogy of Fallot, correlating with FGR and low birthweight.^30^ Consistent with these hemodynamic findings, placental histopathology in women with Fontan physiology or cardiomyopathy shows villous hypoplasia and ischemic changes.^31^^,^^32^ On the other hand, placental angiogenic imbalance itself has been associated with maternal cardiovascular health, both during and after pregnancy. In low-risk pregnancies, lower PlGF concentrations have been associated with subtle systolic impairment (reduced mitral annular s′ velocity) and signs of diastolic dysfunction (larger left atrial area).^33^ Similarly, higher sFlt-1 correlated with concentric remodeling, while an increased sFlt-1/PlGF ratio was related to altered diastolic indices and higher blood pressure.^33^ In HDPs, higher sFlt-1 was associated with increased left ventricular mass, while both elevated sFlt-1 and reduced PlGF were linked to diastolic dysfunction.^34^ Moreover, women with a history of preeclampsia have an increased long-term risk of chronic hypertension, ischemic heart disease, stroke, and heart failure.^35^ Importantly, higher sFlt-1/PlGF ratios have been associated with adverse cardiovascular remodeling and increased cadiovascular risk factors approximately a decade after delivery.^36^ These observations support that placental angiogenic imbalance may reflect an underlying vascular susceptibility that persists beyond pregnancy.^37^

In this context, the increased angiogenic imbalance observed in CHD pregnancies could stem from 2, nonmutually exclusive mechanisms: either as a secondary effect of subtle maternal hemodynamic alterations, or as a manifestation of an intrinsic angiogenic dysregulation. Most studies examining placental angiogenic imbalance in the context of CHD have focused on fetal rather than maternal CHD. Interestingly, these studies report an imbalance between proangiogenic and antiangiogenic placental factors in the serum of mothers carrying a fetus with CHD, characterized by elevated sFlt-1 and decreased PlGF levels.^38^^,^^39^ Llurba et al^38^ demonstrated that this angiogenic imbalance, along with markers of chronic hypoxia, were also present in the fetal cardiac tissue. The authors proposed 2 potential explanations for the abnormal angiogenic pattern observed in pregnant women carrying a fetus with CHD. The first one stipulates that an insufficient trophoblast invasion of the spiral arteries in the mothers leads to placental hypoxia, resulting in placental dysfunction with overproduction of sFlt-1, and simultaneously fetal hypoxia, which could contribute to the development of CHD. Alternatively, CHD in the fetus could stem from an intrinsic disruption of angiogenesis, potentially originating in the trophoblastic stage, impacting both cardiac development and placental function. Indeed, the placenta and the heart are both vascular organs that develop simultaneously during pregnancy, with shared regulatory and signaling pathways.^40^ It is therefore conceivable that an intrinsic vascular dysfunction persisting in adulthood in women with CHD may predispose them to placental dysfunction.

### Association between angiogenic imbalance and adverse outcomes in CHD pregnancies

While the higher risk of obstetric and neonatal complications in CHD pregnancies is well recognized,^1^, ^2^, ^3^, ^4^^,^^10^^,^^41^ the specific pathophysiological mechanisms remain unclear, limiting our ability to predict and manage these outcomes effectively. Our findings suggest that placental angiogenic imbalance may be a key factor contributing to these complications. Specifically, among women with CHD, those with elevated sFlt-1 had a significantly higher incidence of adverse outcomes, whereas those with low PlGF or a high sFlt-1/PlGF ratio showed only a nonsignificant trend toward increased risk. Interestingly, the stronger association with sFlt-1, rather than PlGF, may be explained by the nature of the complications observed in our cohort. Gaccioli et al^14^ demonstrated that in preeclampsia, the elevated sFlt-1/PlGF ratio results mainly from increased placental sFlt-1 production, whereas in FGR, the imbalance is due to reduced placental PlGF. In our CHD cohort, the most common complications were HDPs, and no cases of FGR were observed, as evidenced by the absence of small-for-gestational-age infants. This may explain why low PlGF levels were not significantly associated with adverse outcomes in our study. Moreover, sFlt-1 is the primary driver of maternal symptoms in preeclampsia, leading to endothelial dysfunction and causing hypertension and proteinuria.^11^^,^^12^

Previous studies have shown that sFlt-1 levels rise up to 5 weeks before the onset of preeclampsia symptoms and that early measurement of the sFlt-1/PlGF ratio can predict the development of the disease.^21^^,^^26^^,^^42^ In our study, although not significant within the CHD group alone, the relative increase in sFlt-1 levels between the first and third trimesters was associated with a higher rate of adverse events in the entire cohort. Furthermore, CHD patients with high sFlt-1 levels in the third trimester had a four-fold higher rate of adverse outcomes compared to those with normal levels. These findings suggest the potential of sFlt-1 as a predictive biomarker for identifying CHD patients at higher risk of pregnancy complications. Larger-scale and longitudinal studies of placental angiogenic biomarkers throughout pregnancy in women with CHD are needed to confirm these findings and to further explore their potential role in the early identification of at-risk patients.

### Study Limitations

This study has several limitations. First, it was designed to identify biological markers of placental dysfunction rather than predict adverse outcomes. Therefore, the results should be interpreted as associations, not predictions, especially since biomarkers were measured in the third trimester, possibly after symptom onset. Additionally, the small sample size, particularly in the CHD group, may have limited our ability to detect significant differences in the subanalysis of longitudinal biomarker changes and their association with outcomes. This was a single-center study conducted in Europe, which may limit the generalizability of our findings to other populations. Furthermore, information on ethnicity and socioeconomic status was not available, which limits the ability to account for these potential confounders. Finally, as our study only assessed biomarker levels at the beginning and end of pregnancy, studies with multiple time points throughout pregnancy are needed to confirm our results and assess the predictive value of these biomarkers.

## Conclusions

Pregnant women with CHD exhibited an imbalance in placental angiogenic biomarkers compared to controls, characterized by increased sFlt-1 and reduced PlGF serum levels in the third trimester. This imbalance was associated with an increased risk of adverse obstetric and neonatal outcomes, particularly in women with higher sFlt-1 concentrations. Our findings support the hypothesis that women with CHD are more prone to placental dysfunction, which may contribute to these adverse outcomes. Additionally, these biomarkers could potentially serve as predictive tools for identifying CHD patients at higher risk of pregnancy complications. Further studies are required to confirm these findings and to better understand the molecular mechanisms involved in placental dysfunction in CHD pregnancies. Gaining this understanding could improve early detection and lead to personalized interventions to reduce adverse outcomes in this high-risk population.

Perspectives **COMPETENCY IN MEDICAL KNOWLEDGE:** This study demonstrates that pregnant women with CHD exhibit a placental angiogenic imbalance, with elevated sFlt-1 and reduced PlGF levels in late pregnancy, associated with higher rates of obstetric and neonatal complications. These findings provide further evidence supporting the role of placental dysfunction as a contributor to adverse pregnancy outcomes in women with CHD. This study paves the way for the future validation and use of biomarkers to assess pregnancy risk and guide monitoring strategies in women with CHD.

**TRANSLATIONAL OUTLOOK:** The identification of placental angiogenic imbalance in CHD pregnancies underscores the need for further research into the mechanisms linking maternal CHD to placental dysfunction and adverse pregnancy outcomes. Future studies should evaluate whether angiogenic biomarkers can contribute to risk stratification in this population and explore potential interventions to improve pregnancy outcomes in women with CHD.

## Funding support and author disclosures

This work was supported by grants from the Fondation Damman and the 10.13039/501100011068Fondation Saint-Luc (Brussels, Belgium). The authors have reported that they have no relationships relevant to the contents of this paper to disclose.
