# Supplementary material for: Placental Angiogenic Imbalance and Its Association With Adverse Outcomes in Congenital Heart Disease Pregnancies
Source: JACC Adv. 2025 Nov 4;4(12):102301. doi: 10.1016/j.jacadv.2025.102301 (PMC12637050; doi:10.1016/j.jacadv.2025.102301)
Supplement: Supplemental_Material [file mmc1.pdf]

Supplemental Appendix

Supplemental Tables – page 2

Supplemental Figures – page 6

Supplemental Tables

Table S1. Association between CHD status and angiogenic biomarkers after adjustment for gestational age at third-trimester sampling.

**Dependent variable**	**Independent variables**	**β**	**95% CI**	***P*-value**
log(sFlt-1)	CHD (yes *vs*. no)	0.14	[0.04 to 0.24]	**0.005**
	Gestational age (weeks)	0.03	[0.01 to 0.05]	**0.001**
log(PlGF)	CHD (yes *vs*. no)	-0.16	[-0.31 to -0.01]	**0.034**
	Gestational age (weeks)	-0.05	[-0.08 to -0.02]	**0.003**
log(sFlt-1/PlGF)	CHD (yes *vs*. no)	0.30	[0.10 to 0.50]	**0.004**
	Gestational age (weeks)	0.08	[0.04 to 0.12]	**<0.001**

CHD, congenital heart disease; CI: confidence interval

Table S2. Comparison of third-trimester biomarker levels between CHD and control women matched for gestational age at sampling.

	**ALL**<br>**N=64**	**Controls**<br>**N=32**	**CHD**<br>**N=32**	***P*-value**
**GA at sampling, weeks**	34.2 [33.2–35.7]	33.8 [33.2–35.5]	34.7 [33.2–36.0]	0.432
**sFlt1 (pg/ml)**	2513 [1714–3163]	1992 [1349–2729]	2578 [2095–4493]	**0.006**
**PlGF (pg/ml)**	312 [195–601]	514 [235–670]	276 [164–510]	**0.087**
**sFlt1/PlGF ratio**	6.9 [2.9–18.3]	3.8 [2.3 –12.1]	9.5 [3.8–26.7]	**0.017**

GA, gestational age

Table S3. Biomarker levels comparison between controls and CHD women after exclusion of patients with a history of pre-pregnancy smoking or IVF.

	**ALL**<br>**N=84**	**Controls**<br>**N=61**	**CHD**<br>**N=23**	***P*-value**
**sFLt-1 (pg/ml)**	2168 [1567–3120]	2002 [1451–2799]	2580 [2092–3758]	**0.010**
**PlGF (pg/ml)**	447 [232–719]	505 [282–752]	247 [183–520]	**0.018**
**sFlt-1/PlGF ratio**	4.5 [2.4–11.6]	3.9 [2.0–7.8]	10.8 [3.9–21.3]	**0.003**

Table S4. Mixed-effects models of group × time interaction for sFlt-1 and PlGF (log-transformed).

	**sFlt-1**			**PlGF**		
	β	95% CI	*P*-value	β	95% CI	*P*-value
**Group (CHD *vs.* Control)**	–0.03	[–0.14 to 0.07]	0.527	0.02	[–0.12 to 0.16]	0.777
**Time (T3 *vs.* T1)**	0.13	[0.07 to 0.19]	**<0.001**	1.06	[0.96 to 1.15]	**<0.001**
**Group × Time (Interaction)**	0.19	[0.08 to 0.31]	**0.002**	–0.19	[–0.37 to –0.02]	**0.038**

Estimates are expressed in log-transformed units. Group effect compares CHD vs. controls at baseline (first trimester). Time effect compares third vs. first trimester in controls. The group × time interaction represents the differential change in biomarker levels from the first to the third trimester in CHD compared with controls. CHD, congenital heart disease; CI: confidence interval; T1, first trimester; T3, third trimester.

Supplemental figures

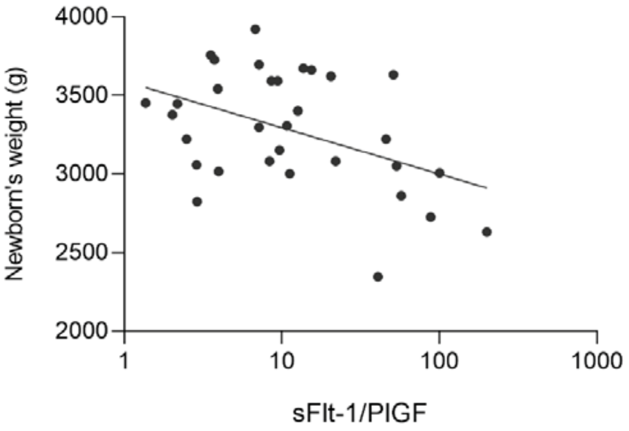

Figure S1. Correlation between the sFlt-1/PlGF ratio and newborn weight in CHD-affected pregnancies. The scatter plot shows an inverse correlation between the sFlt-1/PlGF ratio measured during the third trimester in women with CHD (n=32) and the weight of their newborns (g) (r = -0.44, *P*=0.011).
